# Supplementary material for: Circulating SPINT1 is a biomarker of pregnancies with poor placental function and fetal growth restriction
Source: Nat Commun. 2020 May 15;11:2411. doi: 10.1038/s41467-020-16346-x (PMC7228948; doi:10.1038/s41467-020-16346-x)
Supplement: Supplementary file 1 — Supplementary Information [file 41467_2020_16346_MOESM1_ESM.pdf]

Supplementary Appendix

This appendix has been provided by the authors to give readers additional information about their work.

Supplement to: Kaitu'u-Lino TJ, MacDonald TM et al., Circulating SPINT1 is a biomarker of pregnancies with poor placental function and fetal growth restriction

Supplementary Figures

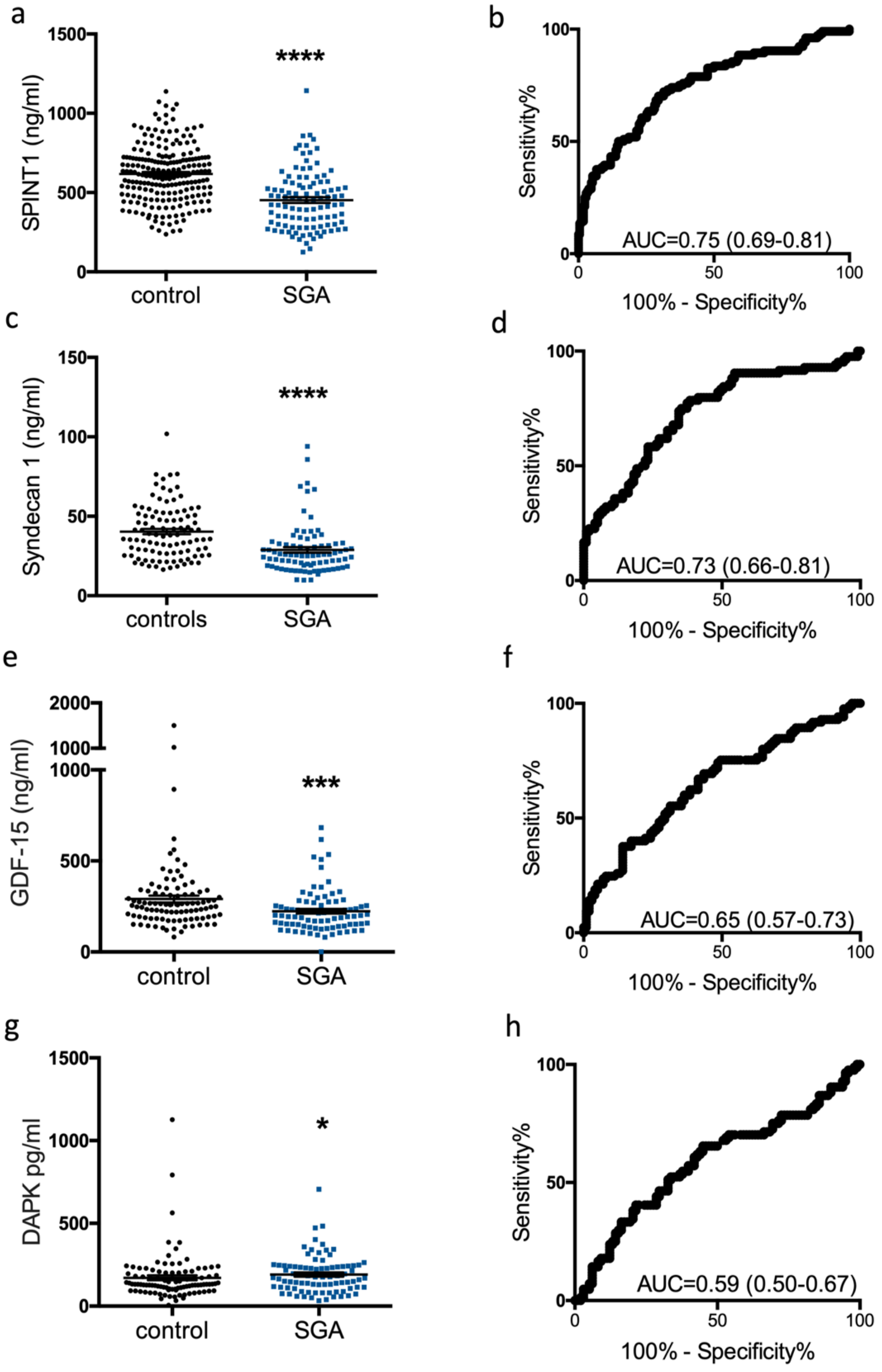

Supplementary Figure 1: Circulating proteins reduced at 36 weeks' gestation among women who will birth a small-for-gestational-age (SGA, birthweight <10th centile) infant. These are four proteins identified from screening 22 new circulating proteins in a case control cohort. SPINT1 (a,b; n= 210 controls, n=104 SGA, p=8.37x10^{-13}), Syndecan-1 (c,d; n=99 controls, n=84 SGA, p=5.13x10^{-8}) and GDF-15 (e,f, n=99 controls, n=85 SGA, p=4.15x10^{-4}) were significantly reduced in the plasma of women destined to deliver an SGA infant. DAPK1 (g,h; n=98 controls, n=84 SGA p=4.60x10^{-2}) was significant increased in the SGA cohort. 105 cases of SGA and 210 matched controls. Individual symbols represent an individual patient. AUC – Area under the ROC curve, with 95% confidence intervals given in brackets. Data in panels a, c, e, g mean± s.e.m *p<0.05, ***p<0.001, ****p<0.0001 using two-tailed Mann-Whitney U tests. Source data included as a source data file.

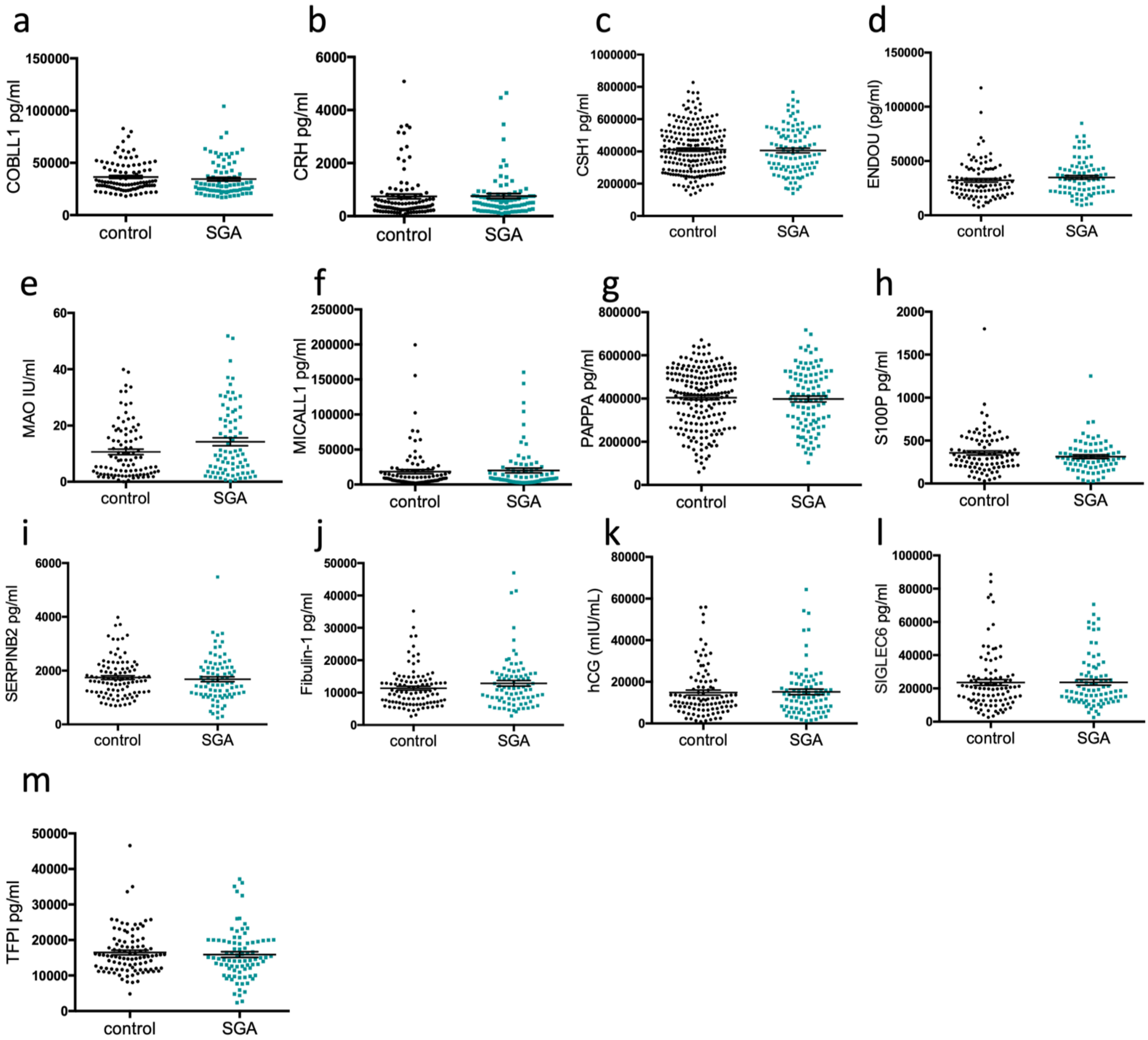

Supplementary Figure 2: Circulating analytes screened at 36 weeks that were detected in plasma but not changed among women destined to deliver a small-for-gestational-age (SGA) infant.

These are 13 of 22 proteins screened in maternal plasma in a case control cohort at 36 weeks' gestation. There were 105 cases of SGA (birthweight <10th centile) and 210 matched controls. Graphed here are proteins we screened that were detectable in the maternal plasma, but were not different in the two groups: COBLL1 (a), CRH (b), CSH1 (c), ENDOU (d), MAO (e), MICALL1 (f), PAPPA (g), S100P (h), SERPINB2 (i), FIBULIN-1 (j), hCG (k), SIGLEC 6 (l) and TFPI (m). Individual symbols represent individual patient analyte levels. The full names of the proteins are listed in supplementary table S3. Source data included as a source data file.

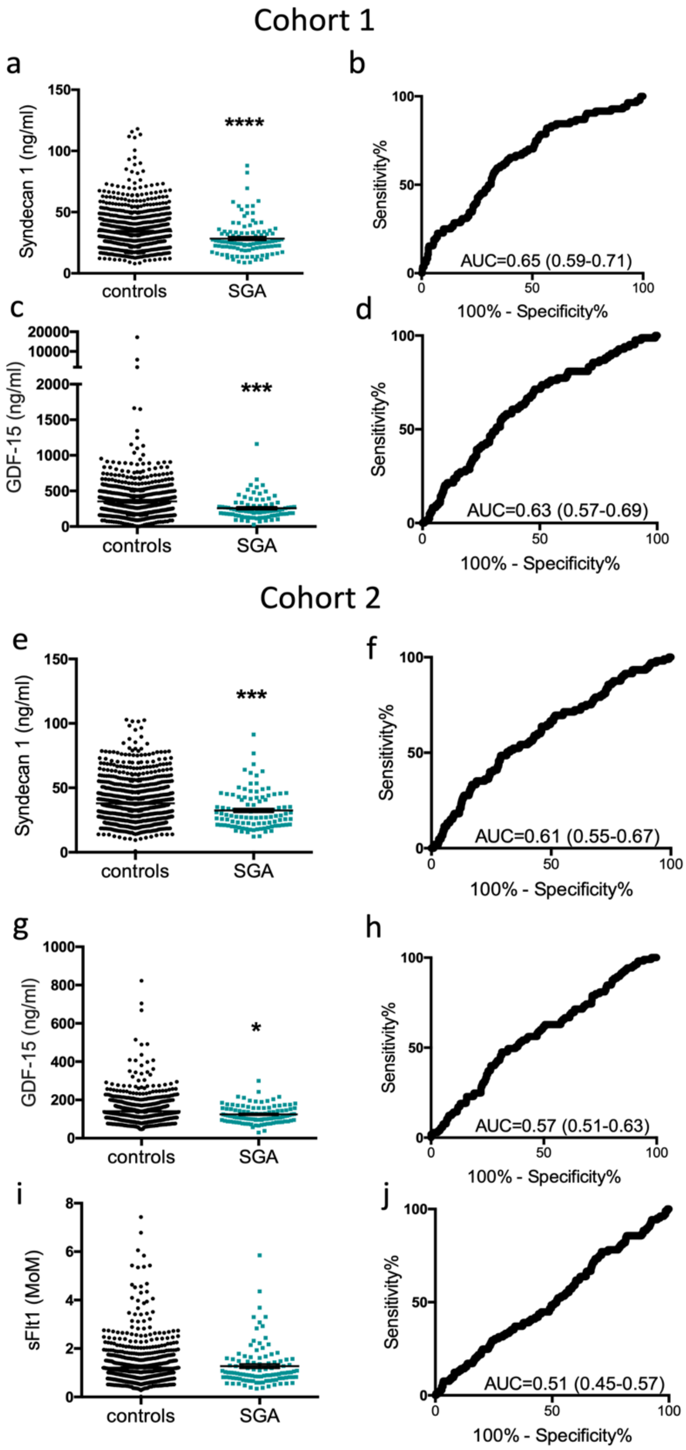

Supplementary Figure 3: Validation of markers in Cohort 1 and 2.
After identifying circulating Syndecan-1 and GDF-15 were significantly altered in the case control cohort at 36 weeks' gestation we assayed both proteins in cohort 1 and 2. We confirmed that maternal plasma concentrations of syndecan-1 (Cohort 1: a,b, $p=1.22x10^{-5}$. Cohort 2 e,f, $p=2.43x10^{-4}$) and GDF-15 (Cohort 1: c,d, $p=1.55x10^{-4}$. Cohort 2: g,h $p=1.68x10^{-2}$) were significantly reduced among women destined to deliver a small-for-gestational-age (SGA, birthweight <10th centile) infant. In cohort 2 there was no significant change in sFlt-1 concentrations in the SGA group (i,j). a,b; n=894 controls, n=106 SGA. c,d; n=895 controls, n=106 SGA. e,f,i,j; n=897 controls, n=105 SGA. g,h; n=893 controls, n=105 SGA. Individual symbols represent an individual patient. AUC – Area under the ROC curve, with 95% confidence intervals given in brackets. Data in panels a, c, e, g, i mean±

s.e.m *$p<0.05$, ***$p<0.001$, ****$p<0.0001$using two-tailed Mann-Whitney U tests. Source data included as a source data file.

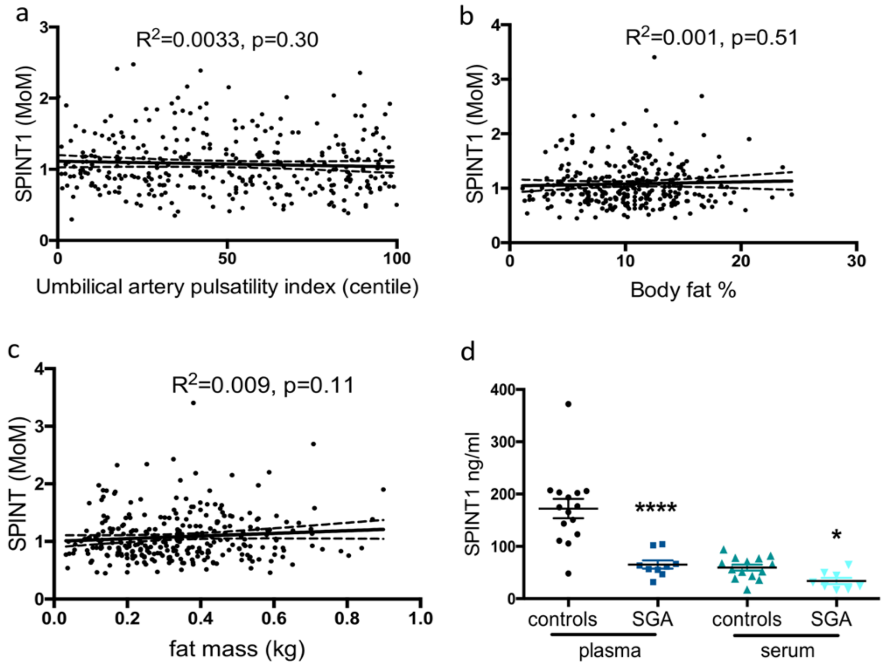

Supplementary Figure 4: SPINT1 levels are not associated with umbilical artery resistance, infant body fat percentage, or fat mass.

A subgroup from the FLAG cohort had antenatal ultrasound measurements, and/or infant body composition assessed after birth using the PEAPOD air displacement plethysmography device. There was no relationship between circulating SPINT1 concentrations at 36 weeks and umbilical artery pulsatility index (a), neonatal body fat percentage (b) or neonatal fat mass (c). We also examined whether SPINT1 concentrations were similar in plasma and serum (d). We measured SPINT1 in women who delivered a <10th centile baby at <34wks relative to levels in healthy controls – where each woman, we obtained both a plasma and serum sample at the same blood draw. While SPINT1 was significantly reduced in the serum of women with SGA, the degree of change was far less than that observed in plasma. Individual symbols represent an individual patient. Panel a; n=327, b,c; n=281, d, data expressed as mean± s.e.m ****p<0.0001, *p<0.05 using two tailed Mann-Whitney U tests vs control. Source data included as a source data file.

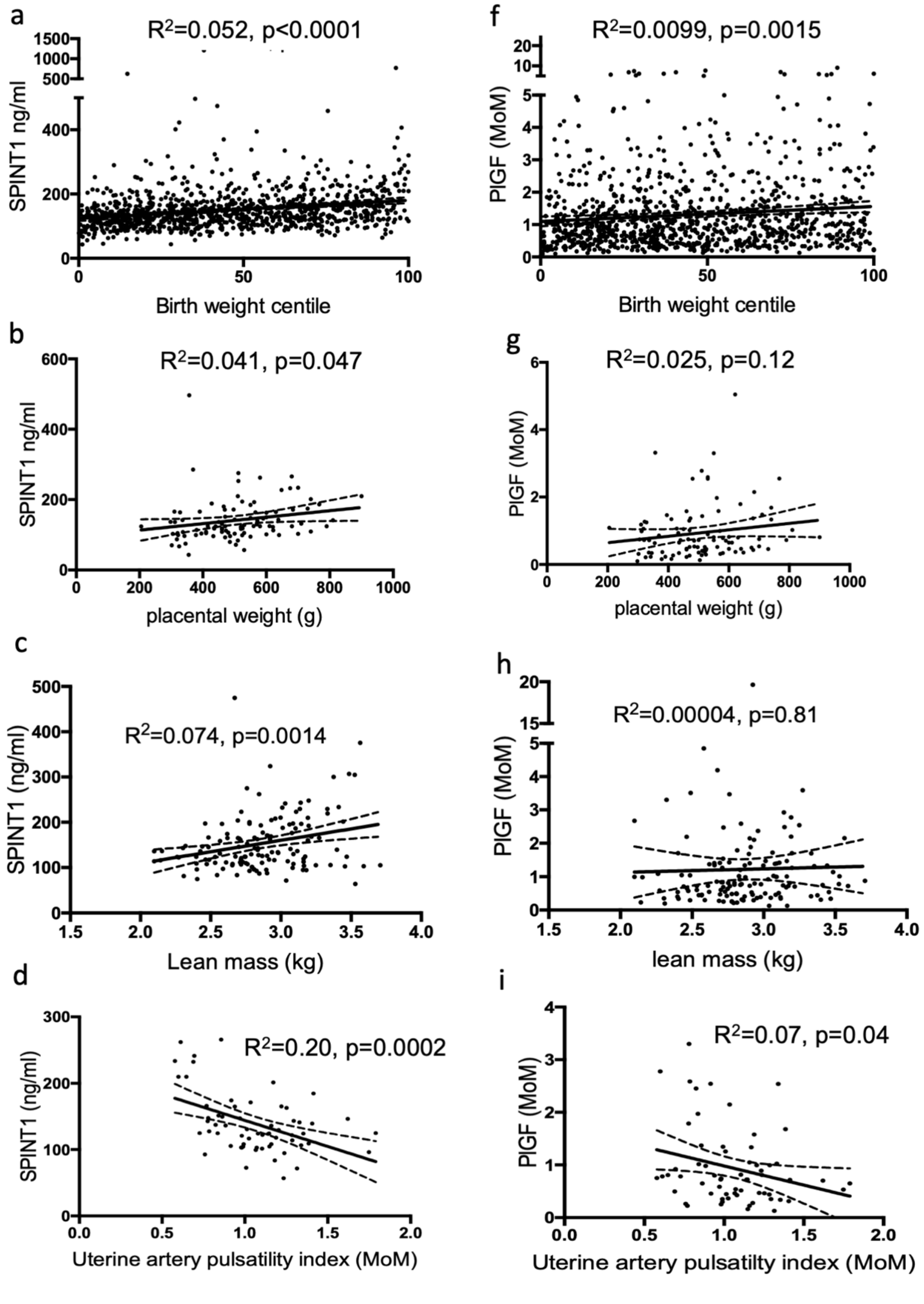

a
R2=0.052, p<0.0001
SPINT1 ng/ml
Birth weight centile
f
R2=0.0099, p=0.0015
PlGF (MoM)
Birth weight centile
b
R2=0.041, p=0.047
SPINT1 ng/ml
placental weight (g)
g
R2=0.025, p=0.12
PlGF (MoM)
placental weight (g)
c
R2=0.074, p=0.0014
SPINT1 (ng/ml)
Lean mass (kg)
h
R2=0.00004, p=0.81
PlGF (MoM)
lean mass (kg)
d
R2=0.20, p=0.0002
SPINT1 (ng/ml)
Uterine artery pulsatility index (MoM)
i
R2=0.07, p=0.04
PlGF (MoM)
Uterine artery pulsatilty index (MoM)

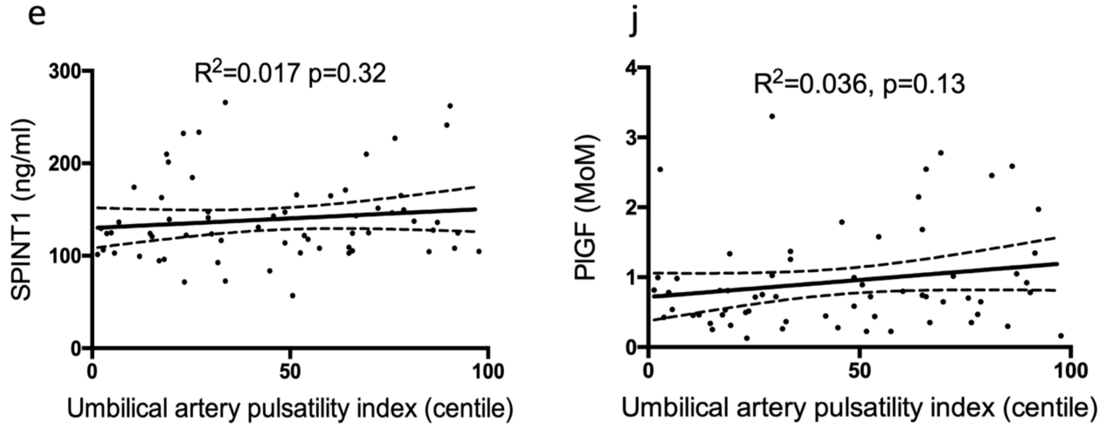

Supplementary Figure 5: Comparing the association between circulating SPINT1 and PlGF at 36 weeks with clinical with markers of placental insufficiency.

These were a subset of participants from the FLAG cohort. Compared to PIGF, circulating SPINT1 concentrations appeared to have a stronger association with birth weight centile (a vs f), placental weight (b vs g), neonatal lean mass (c vs h) and uterine artery resistance (d vs i). Neither were significantly correlated with umbilical artery resistance (e and j). Each datapoint represents an individual patient. a; n=999, f; n=1002, b,g; n=96, c,h; n=136, d, e, i, j; n=63. Source data included as a source data file.

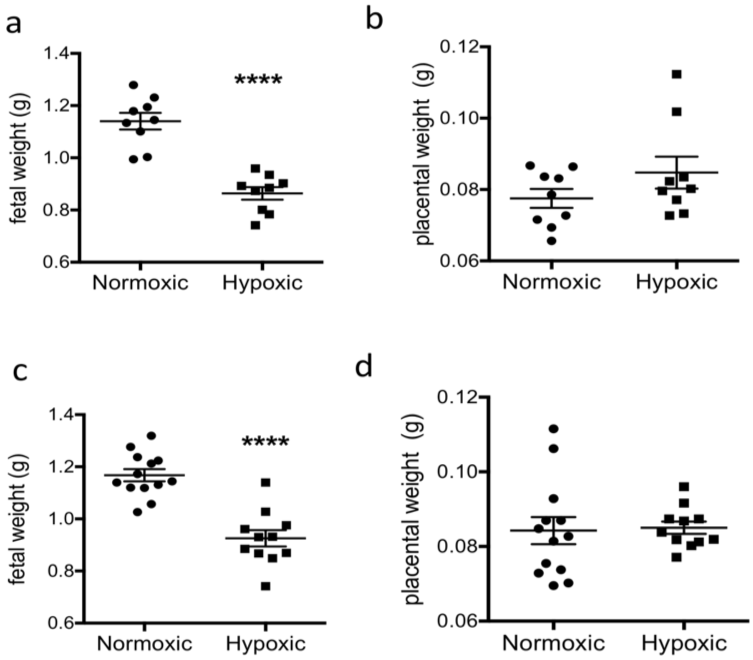

Supplementary Figure 6: Murine fetal, but not placental weights are reduced by maternal exposure to hypoxia.
Following exposure to maternal hypoxia, fetal weight (a; n=9 normoxic, n=9 hypoxic from separate litters. c; n=13 normoxic, n=11 hypoxic from separate litters) but not placental weight (b; n=9 normoxic, n=9 hypoxic from separate litters. d; n=13 normoxic, n=11 hypoxic from separate litters) was significantly reduced. Fetal and placental weights shown in a,b relate to the placentas where SPINT1 mRNA expression was measured (Figure 4e, main manuscript), whilst fetal and placental weights shown in c,d relate to the placentas where SPINT1 protein expression was assessed (Figure 4f, main manuscript). Each individual symbol represents an individual fetus or placenta. Data expressed as mean± s.e.m ****$p<0.0001$ using two-tailed Mann-Whitney U tests. Source data included as a source data file.

	Small-for-gestational-age N=106 (10.6%)	Controls N=895 (89.4%)	*P*
**Age**	32.3 (4.3)	32.6 (4.3)	0.46
**Booking body mass index**	23.8 [21.5 – 28.5]	24.1 [21.7 – 27.4]	0.64
**Nulliparous**	60 (56.6%)	438 (48.9%)	0.15
**Cigarette Smoking**			
- **Current**	9 (8.5%)	23 (2.6%)	0.004
- **Ex-smoker**	26 (24.5%)	203 (22.7%)	
- **Never**	71 (67.0%)	669 (74.7%)	
**Gestational Diabetes Mellitus**	19 (17.9%)	119 (13.2%)	0.18
**Preeclampsia**	7 (6.6%)	32 (3.6%)	0.18
**Onset of labour**			
- **Spontaneous**	38 (35.8%)	444 (49.6%)	0.01
- **Induced**	49 (46.2%)	297 (33.2%)	
- **No labour**	19 (17.9%)	154 (17.2%)	
**Mode of birth**			
- **Normal vaginal birth**	48 (45.3%)	446 (49.8%)	0.62
- **Instrumental delivery**	22 (20.8%)	158 (17.7%)	
- **Caesarean section**	36 (34.0%)	291 (32.5%)	
**Gestation at delivery (weeks^{+days})**	39^{+0} [38^{+0} – 40^{+0}]	39^{+5} [38^{+6} – 40^{+3}]	<0.0001
**Birthweight (g)**	2728 [2448 – 2955]	3510 [3250 – 3800]	<0.0001
**Birthweight centile**	5.4 [2.7 – 8.2]	50.7 [30.0 – 75.0]	<0.0001

Supplementary Table 1: Maternal characteristics and pregnancy outcomes for Cohort 1
Data presented as mean (standard deviation) if normally distributed data, as median [25th – 75th percentile] if not normally distributed data, and as number (%) if categorical. Small-for-gestational-age defined as birthweight <10th centile

	Small for gestational age n=105 (10.5%)	Controls n=897 (89.5%)	*P*
**Age**	33.2 (4.2)	32.3 (4.1)	0.04
**Booking body mass index**	24.7 [22.1 – 28.7]	24.4 [22.0 – 27.7]	0.11
**Nulliparous**	44 (41.9%)	399 (44.5%)	0.68
**Cigarette Smoking**			
- **Current**	9 (8.6%)	22 (2.5%)	0.002
- **Ex-smoker**	20 (19.0%)	208 (23.2%)	
- **Never**	76 (72.4%)	667 (74.4%)	
**Gestational Diabetes Mellitus**	9 (8.6%)	114 (12.7%)	0.27
**Preeclampsia**	7 (6.7%)	39 (4.3%)	0.32
**Onset of labour**			
- **Spontaneous**	42 (40.0%)	407 (45.4%)	0.51
- **Induced**	42 (40.0%)	312 (34.8%)	
- **No labour**	21 (20.0%)	178 (19.8%)	
**Mode of Birth**			
- **Normal vaginal birth**	54 (51.4%)	437 (48.7%)	0.21
- **Instrumental delivery**	11 (10.5%)	154 (17.2%)	
- **Caesarean section**	40 (38.1%)	306 (34.1%)	
**Gestation at delivery (weeks^{+days})**	39^{+1} (1^{+2})	39^{+3} (1^{+1})	0.02
**Birthweight (g)**	2750 [2590 – 2930]	3450 [3190 – 3740]	<0.0001
**Birthweight centile**	5.4 [3.0 – 7.5]	46.9 [28.3 – 70.6]	<0.0001

Supplementary Table 2: Maternal characteristics and pregnancy outcomes for Cohort 2
Data presented as mean (standard deviation) if normally distributed data, as median [25th – 75th percentile] if not normally distributed data, and as number (%) if categorical. Small-for-gestational-age defined as birthweight <10th centile.

Protein	Company	Inter-assay CV	Dilution (36 wks)	Detection Range (pg/ml or as indicated)
SPINT1	Sigma	<12%	1:6	819 - 200,000
SYNDECAN-1	Thermo Fisher	<12%	1:35	16 - 4000
sFlt-1	Roche	<5%	neat	10-85000
PlGF	Roche	<5%	neat	3-10000
GDF-15	Thermo Fisher	<12%	1:2000	1.1 - 800
DAPK1	MyBioSource	<10%	neat	31 - 2000
COBLL1	MyBioSource	<10%	neat	5000 - 100,000
CRH	MyBioSource	<12%	1:5	15.6 - 1000
CSH1	LifeSpan BioSciences	<12%	1:10	2500 – 160,000
ENDOU	EIAab Science	<10.4%	1:5	15.6 - 1000
MAO	Cusabio	<10%	1:2	0.9 - 60IU/ml
MICALL1	MyBioSource	<15%	neat	3120 -100,000
PAPP-A	R & D Systems	Not found	1:12	781 -50,000
S100P	MyBioSource	<10%	1:2 or neat	78 - 5000
SERBINB2	MyBioSource	<12%	1:50	15.6 - 1000
FIBULIN-1	EIAab	≤9.4%	1:4	470 - 30,000
hCG	ALPCO Diagnostics	≤6.9%	1:200	5 - 300mIU/ml
SIGLEC-6	R & D Systems	Not found	1:8	156 – 10,000
TFPI	R & D Systems	Not found	1:350	15.6 - 1000
GM2A	MyBioSource	<10%	not detectable	15.6 - 1000
KISS1	Aviva Systems	≤5.9%	most samples not detectable	156 – 10,000
PVRL3	MyBioSource	<12%	most samples not detectable	156 – 10,000
PSG6	MyBioSource	<10%	not detectable	312 – 20,000
PTPRF	CUSABIO	<10%	not detectable	6.25 - 400

Supplementary Table 3: Circulating proteins measured and the Commercial ELISAs used. Commercially available ELISAs were sourced to measure proteins of interest.

sFlt-1 = soluble FMS-like tyrosine kinase-1

PlGF = Placental growth factor

GDF-15 = Growth differentiation factor-15

DAPK1 = Death associated protein kinase

COBLL1 = Cordon-Bleu protein Like 1

CRH1 = corticotropin releasing hormone1

ENDOU = endonuclease, poly(U) specific

MAO = Monoamine oxidase A

MICALL-1 = MICAL-like protein 1

PAPP-A = pregnancy-associated plasma protein-A

S100P = S100 calcium-binding protein P

SerbinB2 = PAI2 = Plasminogen activator inhibitor-2
hCG = human chorionic gonadotrophin
SIGLEC6 = Sialic acid binding Ig like lectin-6
TFPI = Tissue factor pathway inhibitor
GM2A = GM2 ganglioside activator
KISS1 = kisspeptin-1
PVRL3 = poliovirus receptor-related 3
PSG6 = pregnancy specific beta-1-glycoprotein 6
PTPRF = protein tyrosine phosphatase receptor type F

	Birthweight <3rd centile	Birthweight <5th centile	Birthweight <10th centile	Birthweight <20th centile	Birthweight <5th centile and nursery admission
**Positive Predictive Value**	10.1%	14.3%	19.7%	15.7%	1.0%
**Negative Predictive Value**	98.3%	96.4%	90.7%	90.0%	99.2%
**Risk ratio (95% CI)**	6.0 (2.8-12.7)	4.0 (2.3-7.0)	3.1 (2.0-4.7)	1.6 (1.1-2.6)	1.3 (0.2-10.3)
**Sensitivity**	42.3%	32.7%	21.7%	32.5%	12.5%
**Specificity**	90.0%	89.9%	89.5%	77.7%	89.9%

Supplementary Table 4: Diagnostic performance of circulating plasma placental growth factor (PIGF) concentration at 36 weeks to predict infants born at different birthweight thresholds.

Model (n = 967)	ROC area (95%CI)	Sensitivity at 90% specificity	LR +	LR -
**<10th centile birthweight:**				
Spint1 alone	0.66 (0.61 – 0.72)	24.5	2.46	0.84
Spint1 adjusted for clinical factors	0.66 (0.61 – 0.72)	22.7	2.26	0.86
Clinical factors only	0.57 (0.51 – 0.63)	14.4	1.44	0.95
**<5th centile birthweight:**				
Spint1 alone	0.71 (0.62 – 0.79)	36.7	3.71	0.70
Spint1 adjusted for clinical factors	0.70 (0.62 – 0.79)	37.2	3.74	0.70
Clinical factors only	0.64 (0.55 – 0.72)	23.3	2.33	0.85
**<3th centile birthweight:**				
Spint1 alone	0.75 (0.64 – 0.85)	42.3	4.23	0.64
Spint1 adjusted for clinical factors	0.76 (0.67 – 0.85)	41.7	4.18	0.65
Clinical factors only	0.69 (0.59 – 0.78)	25.0	2.51	0.83

Supplementary Table 5: Diagnostic performance of circulating SPINT1 at 36 weeks' gestation to predict <10th, 5th and <3rd centile birthweights with adjustments for maternal clinical factors. Clinical factors adjusted for were age, smoking, parity and Body mass Index. Analysis was performed using logistic regression analysis. Analysis was done on cohort 2. Total numbers where all clinical variables were available for analyses were n = 967. Numbers vary to those presented in table 1 (n=998) because of missing maternal characteristics for some patients. ROC – Receiver Operated Curve. CI – Confidence Interval. LR – Likelihood ratio.

**Predicting <10th centile birthweight**	**ROC area (95%CI)**	**Sensitivity at 90% specificity**	**LR +**	**LR -**
**Logistic regression**				
Spint1 + GDF15 + PIGF + Syndecan	0.67 (0.62 – 0.73)	27.4	2.76	0.81
Spint1 + GDF15	0.66 (0.61 – 0.72)	26.4	2.67	0.82
**Spint1**	**0.66 (0.61 – 0.72)**	**24.5**	**2.46**	**0.84**
GDF15	0.57 (0.52 – 0.63)	13.2	1.33	0.96
PIGF	0.59 (0.53 – 0.65)	21.7	2.17	0.87
Syndecan	0.61 (0.55 – 0.67)	17.9	1.80	0.91
**Lasso [1]**				
Spint1 + GDF15 + PIGF + Syndecan	0.67 (0.62 – 0.73)	28.3	2.86	0.80

Supplementary Table 6: Diagnostic performance of circulating plasma SPINT1, GDF15, PlGF and Syndecan-1 concentrations at 36 weeks to predict infants born at <10th centile birthweight.
[1] lambda = 0.0018269, no variable dropped. ROC – Receiver Operated Curve. CI – Confidence Interval. LR – Likelihood ratio.

**Predicting <5th centile birthweight**	**ROC area (95%CI)**	**Sensitivity at 90% specificity**	**LR +**	**LR -**
**Logistic regression**				
Spint1 + GDF15 + PIGF + Syndecan	0.72 (0.65 – 0.80)	34.7	3.49	0.73
Spint1 + gdf15	0.71 (0.63– 0.79)	38.8	3.90	0.68
**Spint1**	**0.71 (0.62 – 0.79)**	**36.7**	**3.71**	**0.70**
GDF15	0.63 (0.54 – 0.71)	16.3	1.64	0.93
PIGF	0.62 (0.53 – 0.71)	32.7	3.30	0.75
Syndecan	0.63 (0.55 – 0.71)	16.3	1.65	0.93
**Lasso [1]**				
Spint1 + GDF15 + PIGF + Syndecan	0.72 (0.65 – 0.80)	34.7	3.49	0.73

Supplementary Table 7: Diagnostic performance of circulating plasma SPINT1, GDF15, PlGF and Syndecan-1 concentrations at 36 weeks to predict infants born at <5th centile birthweight. [1]lambda = 0.0019988, no variable dropped. ROC – Receiver Operated Curve. CI – Confidence Interval. LR – Likelihood ratio.

**Predicting <3th centile birthweight**	**ROC area (95%CI)**	**Sensitivity at 90% specificity**	**LR +**	**LR -**
**Logistic regression**				
Spint1 + GDF15 + PIGF + Syndecan	0.78 (0.68 – 0.88)	50.0	5.04	0.56
Spint1 + GDF15	0.75 (0.64– 0.85)	42.3	4.27	0.64
**Spint1**	**0.75 (0.64 – 0.85)**	**42.3**	**4.24**	**0.64**
GDF15	0.62 (0.50 – 0.74)	15.4	1.55	0.94
PIGF	0.69 (0.57 – 0.80)	42.3	4.28	0.64
Syndecan	0.66 (0.56 – 0.77)	15.4	1.56	0.94
**Lasso [1]**				
Spint1 + GDF15 + PIGF + Syndecan	0.78 (0.68 – 0.88)	50.0	5.04	0.56

Supplementary Table 8: Diagnostic performance of circulating plasma SPINT1, GDF15, PlGF and Syndecan-1 concentrations at 36 weeks to predict infants born at <3rd centile birthweight.
[1] lambda = 0.0008931, no variable dropped. ROC – Receiver Operated Curve. CI – Confidence Interval. LR – Likelihood ratio.

	Birthweight < 3rd centile	Birthweight < 5th centile	Birthweight < 10th centile	Birthweight < 20th centile	Birthweight < 5rd centile and nursery admission
**Positive Predictive Value**	4.0%	8.7%	14.4%	24.3	1.0%
**Negative Predictive Value**	97.5%	95.5%	89.8%	76.4	99.2%
**Risk Ratio (95%CI)**	1.61 (0.57 – 4.59)	1.96 (0.98 – 3.91)	1.56 (0.94 – 2.59)	1.02 (0.72 – 1.48)	1.27 (0.16 – 10.21)
**Sensitivity**	15.4%	18.4%	14.2%	10.6%	12.5%
**Specificity**	90.0%	90.1%	90.0%	89.8%	89.9%

Supplementary Table 9: Diagnostic performance of the sFlt/PIGF ratio in cohort 2. Expressing the data as a PIGF/sFlt ratio provides identical performance characteristics.

	**Small-for-gestational-age n=83 (28.5%)**	**No SGA n=208 (71.5%)**	***P***	***MAViS cohort (n=518)***
**Age**	34.2 (5.6)	34.5 (4.9)	0.65	34.5 (5.4)
**Booking body mass index**	29.1 [24.8 – 33.9]	29.7 [25.3 – 34.4]	0.54	29.6 [25.6-34.3]
**Nulliparous**<br>- **Multiparous (no prior history)[§]**<br>- **Multiparous (prior history)**	23 (27.7%)<br>17 (20.5%)<br>43 (51.8%)	61 (29.3%)<br>63 (30.3%)<br>84 (40.4)	0.20	159 (30.6%)<br>137 (26.5%)<br>222 (42.9%)
**Cigarette Smoking (current)**	3/71 (4.2%)	5/189 (2.7%)	0.51	25/458 (5.4%)
**Hypertension**<br>- **Chronic hypertension**<br>- **Renal hypertension**	58 (69.9.3%)<br>8 (9.6%)	142 (68.3%)<br>33 (15.9%)	0.30	345 (66.6%)<br>67 (12.9%)
**Diabetes Mellitus**<br>- **Type 1**<br>- **Type 2**<br>- **Gestational DM**<br>- **Not tested for GDM**	0 (0%)<br>3 (3.6%)<br>13 (15.7%)<br>20 (24.1)	8 (3.8%)<br>9 (4.3%)<br>29 (13.9%)<br>44 (21.2%)	0.46	18 (3.5%)<br>36 (7.0%<br>64 (12.4%)<br>144 (27.8%)
**Preeclampsia**<br>- **<34 weeks**<br>- ***<37 weeks (inclusive <34)***<br>- **>37 weeks**	13 (15.7%)<br>*19 (22.9%)*<br>7 (8.4%)	5 (2.4%)<br>*15 (7.2%)*<br>17 (8.2%)	<0.001	44 (8.5%)<br>74 (14.3%)<br>30 (5.8%)
**SGA**<br>- **<10th centile**<br>- **<5th centile**	83 (100%)<br>43 (52%)	0 (0%)<br>0 (0%)	n/a	142/510[#] (27.8%)<br>75/510 (14.4%)
**Onset of labour**<br>- **Spontaneous**<br>- **Induced**<br>- **No labour**<br>- **Not recorded**	5 (6.0%)<br>41 (49.5%)<br>33 (39.8%)<br>3 (3.6%)	29 (13.9%)<br>98 (47.1%)<br>75 (36.1%)<br>6 (2.9%)	0.19	59 (11.4%)<br>218 (42.1%)<br>205 (39.6%)<br>36 (6.9%)
**Mode of birth**<br>- **Normal vaginal birth**<br>- **Instrumental delivery**<br>- **Caesarean section**	29 (34.9%)<br>6 (7.2%)<br>48 (57.8%)	84 (40.5%)<br>21 (10.5%)<br>102 (49.0%)	0.44	198 (38.6%)<br>37 (7.1%)<br>273 (52.8%)
**Delivery**<br>- **<37 weeks**<br>- **<34 weeks**	30 (36.1%)<br>13 (15.7%)	23 (11.1%)<br>5 (2.4%)	<0.001	152/510[#] (29.8%)<br>62/510 (12.2%)
**Gestation at birth (weeks^{+days})**	37^{+3} [36^{+0} – 38^{+3}]	38^{+3} [37^{+5} – 39^{+1}]	<0.001	38 [36^{+5}-38^{+6}]

**Birthweight (g)**	2551 [2100 – 2680]	3280 [2990 – 3530]	<0.001	2980 [2552-3355]
**Birthweight centile**	4.98 [2.27 – 7.74]	44.72 [26.02 – 66.11]	<0.001	27.88 [8.82-57.01]

Supplementary Table 10: Maternal characteristics and pregnancy outcomes for the Manchester Antenatal Vascular Service (MAViS) case-cohort study (n=291) included in this study. The last column shows the clinical characteristics of the entire MAViS cohort from which these samples were (n=518).

§ Prior history of pre-eclampsia/placental disease (small for gestational age, pre-eclampsia, abruption, stillbirth with evidence of placental insufficiency)
# Outcome data not available for 8 women.

	Preterm controls born with a birthweight >10th centile (n=23)	Preterm fetal growth restriction (n=13)
**Maternal Age** (yrs)	32.1 [26.0-36.4]	29.9 [23.7-33.4]
**Gestation at Delivery**(weeks)	30.0 [28.7-31.9]	31.4 [30.4-32.3]
**Body mass index** (kg/m^2)	27.3 [24.2-29.8]	23.0 [19.0-29.0]
**Parity** no. (%) *		
0	7 (30)	10 (77)
1	10 (43)	1 (8)
≥2	6 (26)	2 (15)
**SBP at Delivery** (mmHg)	120 [113-130]	120 [117-130]
**DBP at Delivery** (mmHg)	70 [70-80]	80 [70-83]
**Birthweight** (g) *	1540 [1226-1823]	999 [870-1126]
**Male** Number (%)	11 (48)	8 (62)

Supplementary Table 11: Clinical characteristics of a preterm fetal growth restriction cohort. These were cases where placental samples were collected from women delivered <34 weeks gestation with preterm fetal growth restriction (birthweight <10th centile), and preterm controls (birthweight >10th centile).

BMI = body mass index, SBP = systolic blood pressure and DBP = diastolic blood pressure. BMI data missing for 5/23 preterm controls. SBP at booking data missing for 11/23 preterm controls and 3/13 IUGR cases. 1/23 control and 1/13 FGR did not provide a read in initial western blot batch run and therefore are missing in Figure 4b. Presented as median [25th – 75th percentile], median (minimum, maximum) or number (%). Two tailed Mann-Whitney U tests used to compare continuous variables, Chi-squared for dichotomous outcomes. *p<0.05 **p<0.001

	Preterm controls born with a birthweight >10th centile (n=15)	Preterm fetal growth restriction (n=9)
**Maternal Age** (years)	32.0 [29.6 – 33.7]	33.5 [31.3 – 38.4]
**Gestation at Delivery***** (weeks)	39.6 [38.6 – 40.1]	28.6 [27.9 – 29.7]
**Gestation at Blood collection** (weeks)	29.7 [26.9 – 30.7]	28.6 [27.4 – 29.1]
**BMI** (kg/m^2)	25.5 [23.5 – 28.0]	32.0 [27.1 – 34.4]
**Parity*** no. (%)		
0	4 (27)	7 (78)
1	8 (53)	1 (11)
≥2	3 (20)	1 (11)
**SBP at Delivery*** (mmHg)	125 [120 – 128]	170 [155 – 175]
**DBP at Delivery*** (mmHg)	79 [72 – 80]	95 [90-100]
**Birth weight** (g) ***	3510 [3220 – 3805]	653 [623 – 859]
**Male** Number (%)	5 (33)	3 (33)

Supplementary Table 12: Clinical characteristics of cases where plasma samples were collected from women who eventually delivered <34 weeks' gestation with preterm fetal growth restriction (birthweight <10th centile), and preterm controls (birthweight >10th centile).
BMI = body mass index, SBP = systolic blood pressure and DBP = diastolic blood pressure. Presented as median [25th – 75th percentile], median (minimum, maximum) or number (%). Two-tailed Mann-Whitney U tests used to compare continuous variables, Chi-squared for dichotomous outcomes.
*p<0.05, ***p<0.001

Supplementary Methods

Discovering circulating biomarkers of placental insufficiency

To identify new biomarkers of placental insufficiency we performed the Fetal Longitudinal Assessment of Growth (FLAG) study. This included prospective collection of blood samples from pregnant women at 28 ($27^{+0} – 29^{+0}$) and 36 ($35^{+0} – 37^{+0}$ days) weeks' gestation from 2015 participants. The FLAG study was undertaken at Mercy Hospital for Women, a tertiary referral hospital in Melbourne Australia. This study was approved by the Mercy Health Research Ethics Committee (Ethics Approval Number R14/12) and written informed consent was obtained from all participants. Identification of pregnancies that ended in the birth of a neonate that was small for gestational age (birthweight centile <10th) was pre-specified as the primary outcome in the study protocol.

We also recruited a sub-cohort consisting of 347 nulliparous participants chosen from those enrolled in the FLAG study to undergo more intensive studies. For this sub-cohort, we performed ultrasound assessments at 28 and 36 weeks' gestation, where several parameters were measured including Doppler assessment of blood flow resistance in the uterine, umbilical and the fetal middle cerebral arteries. Post birth, where possible, we measured neonatal body composition (lean body mass and fat mass) within 4 days of birth by performing air displacement plethysmography studies using a PEAPOD device (COSMED, Concord, CA, USA). Further methods for recruitment for the FLAG study are described below.

To identify new biomarkers of placental insufficiency near term gestation we focused on the 36 week samples, where 1996 plasma samples were available. We divided the cohort approximately in half to discover, and then subsequently validate markers. Samples from the first 997 consecutively recruited participants constituted Cohort 1 and those from the second 998 consecutively recruited participants constituted Cohort 2.

We initially screened 22 circulating proteins in a 1:2 nested case (105 neonates born small-for-gestational-age, SGA, birthweight <10th centile) control (210 neonates born with a birthweight ≥10th centile) set selected from Cohort 1. The controls were group-matched to cases for maternal age, booking body mass index, smoking status, gestational diabetes mellitus, and parity.

We selected proteins that are highly expressed in placenta relative to other tissues by referencing two bioinformatic databases. We selected proteins that were both 1) highly expressed at the mRNA level in placenta relative to all other human tissues using BioGPS (Biogps.org); and 2) abundantly expressed in its protein form on the membrane surface of the placenta (using Protein Atlas [www.proteinatlas.org]). The function of many of these proteins remain poorly understood. However, we postulated that because these are highly expressed in the placenta, most will play important biological roles and the expression of some may be perturbed in the presence of placental insufficiency.

The proteins screened where circulating concentrations were different among cases of SGA compared to controls were re-assayed in a new batch assay run on all samples from Cohort 1. Those that remained significantly associated with SGA were then assayed in Cohort 2. We also measured placental growth factor (PIGF) and soluble fms-like tyrosine kinase-1 in Cohort 2. Further methodology detailing how the proteins were measured is included below.

Developing diagnostic tests for placental insufficiency

The intent of the FLAG study was to examine whether a combination of these markers was better at identifying fetal growth restriction, compared to one in isolation. We examined the diagnostic

performance of potential markers, either alone or in combination, to predict neonates born at birthweight centiles; <20th, <10th, <5th and <3rd; and those with birthweight <5th centile who required nursery admission (the latter is a small cohort likely to have suffered significant placental insufficiency).

We generated potential diagnostic tests by examining whether we could combine the different proteins in results obtained in Cohort 2. We tried to develop modelling by setting the specificity at around 90%, which would equate to a 10% screen positive rate. We found SPINT1 performed the best and none of the other markers added to its performance. Therefore, we validated the diagnostic performance of SPINT1 in Cohort 1. To adjust for the fact that the research ELISA we used exhibited variability in reporting the absolute SPINT1 concentrations between the batches run for Cohorts 1 and 2, we expressed SPINT1 results as multiples of the median (MoMs).

We also developed (Cohort 2) a 4-tier model of risk for these different low birthweight ranges based on different SPINT1 MoM cut off levels. These cut-off levels were arbitrarily chosen when developing the test in Cohort 2.

Further methods on describing the statistical analyses are detailed below.

SPINT1 and other parameters of placental insufficiency

We further investigated SPINT1 by correlating circulating levels with several other clinical indicators of placental insufficiency using data obtained from our sub-cohort study. We also measured circulating SPINT1 concentrations and placental expression in a separate cohort with fetal growth restriction delivered at <34 weeks gestation, where we performed further laboratory investigations in vitro, and in a mouse model of fetal growth restriction. Detailed methods on the laboratory studies are described below.

Recruitment of samples for the FLAG cohort

Women were screened for eligibility and were invited to participate at their oral glucose tolerance test, universally offered around 28 weeks' gestation to test for gestational diabetes mellitus. Inclusion Criteria - English-speaking women aged over 18 years, with a singleton pregnancy and normal mid-trimester fetal morphology examination were eligible to participate. Samples from women where a SGA fetus was suspected at the time of blood sampling were not excluded. Exclusion criteria – multi-gestation pregnancies or pregnancies identified as having fetal anomalies.

Maternal blood sample collection and preparation for the FLAG cohort

Participants donated blood samples at between 27^{+0} to 29^{+0} weeks' and/or 35^{+0} to 37^{+0} weeks' gestation inclusive. Whole blood was collected in a 10ml ethylenediaminetetraacetic acid tube. Plasma was stored at -80°C until the time of sample analysis.

Outcomes and definitions of cases for the FLAG cohort

Maternal characteristics and pregnancy outcomes were obtained from review of each participant's medical record, investigation results and hospital database entry, by a single clinician, blinded to any protein levels.

Birthweight centile calculations
Infant birthweights were assigned a customised centile using the GROW software[1] (www.gestation.net), which generates a 'term optimal weight' based on an optimised fetal weight standard. We adjusted for the following non-pathological factors: maternal height, weight and parity; infant sex; and exact gestational age. Coefficients for the Australian dataset of GROW were informed by a local dataset; the multiple regression model has a constant to which weight is added or subtracted for each of the adjusted variables. SGA was defined as customised birthweight <10th centile. We compared the circulating protein levels among SGA cases, to those of the controls.
We determined *a priori* plans to further investigate our most promising biomarkers for their association with several clinical parameters associated with placental insufficiency and fetal growth restriction by: (i) validating our findings in the entire first 1000 sample cohort; (ii) validating our findings in the second 1000 sample cohort; (iii) investigating the predictive potential of the biomarker in the 28 week blood samples; (iv) correlating candidate biomarkers with 36 week Doppler ultrasound parameters that are antenatal indicators of uteroplacental function; and (v) correlating candidate biomarkers with neonatal body composition measures – indicators of in utero nutrient supply.

Doppler ultrasound parameters at 36 weeks
Some nulliparous participants were also involved in the ultrasound-based arm of the FLAG study. For this, 347 women underwent a 36 (35^{+0}-37^{+0}) week ultrasound assessment where transabdominal colour and pulsed-wave Doppler were used to measure the mean maternal uterine artery pulsatility index (PI) and the umbilical artery PI. Measurements were taken during periods of fetal apnoea and inactivity with the angle of insonation close to zero. The umbilical artery PI was measured in a free loop of umbilical cord away from cord insertion sites. For the maternal uterine artery the probe was placed in each of the iliac fossae, and the waveform recorded within 1cm of the uterine artery crossing the external iliac artery[2]. PI values were measured in triplicate and the mean calculated. Average uterine artery PI values were obtained for both the right and left vessels, and these averaged to provide the overall mean PI. For each of the PI values, the gestation-dependent centile (if normally distributed), or the multiples of the median (MoM) were determined. Treating clinicians were blinded to the uterine artery PI results.

Inclusion and exclusion criteria for samples from the MAViS clinic

To validate the observation that SPINT1 is associated with placental insufficiency we measured SPINT1 in a high-risk cohort from the United Kingdom – the Manchester Antenatal Vascular Service (MAViS clinic). Women gave written informed consent to donate samples for future research studies. The study was approved by the NRES Committee North West 11/NW/0426.

The inclusion criteria for women in the MAViS study were: 1. chronic hypertension BP ≥140/90 at ≤20 weeks; 2. chronic hypertension requiring antihypertensive treatment ≤ 20 weeks; 3. pre gestational diabetes with evidence of vascular complications (hypertension, nephropathy); 4. history of ischeamic heart disease and 5. previous early onset preeclampsia.

Prespecified outcomes in the protocol for the MAViS biobank collection: primary outcome was the development of pregnancy complication requiring preterm birth (<37 weeks), secondary outcomes were the development of preeclampsia, birth of a small for gestational age neonate and early preterm birth (<34 weeks' gestation).

For our validation study cases and controls were selected from the MAViS biobank to specifically address whether SPINT1 was differentially expressed in pregnancies destined to deliver a neonate

<10th centile birthweight (see supplementary table 10). Hence, for the purposes of our present study, the delivery of a small for gestational age was our primary prespecified outcome.

Recruitment and collection of samples from women with preterm fetal growth restriction

Blood samples or placental samples were obtained from women with a diagnosis of preterm fetal growth restriction and were delivered at <34 weeks' gestation, or gestation matched controls. Sample collection as part of the Mercy Tissue Bank was approved by the Mercy Health Research Ethics Committee (Ethics Approval Number R11/34) and written informed consent was obtained from all participants. Use of samples for this study was approved by the Mercy Health Research Ethics Committee (Ethics approval number R18/55). In this cohort fetal growth restriction <34 weeks' gestation was pre-specified as the primary outcome.

For the fetal growth restriction cohort, women were invited to participate when it was diagnosed on ultrasound. We also only included their samples if they were delivered <34 weeks' gestation and growth restriction was confirmed following birth (by the fact that the neonatal weight was <10th centile).

Controls were women who consented to blood collection at matched gestations but went on to deliver a normal sized infant (>10th centile) at term gestation.

For the control group for the preterm placental samples we obtained placentas from women who were delivered for reasons other than fetal growth restriction or hypertensive diseases (the mother remained normotensive), such as vasa praevia, maternal medical conditions, spontaneous preterm labour or antepartum haemorrhage.

For the placental studies all women were delivered by caesarean section for both the fetal growth restriction and control cohorts.

Whole blood was collected in a 10ml ethylenediaminetetraacetic acid tube. Plasma was stored at -80°C until the time of sample analysis. Placental tissue was obtained immediately following delivery. Maternal and fetal surfaces were removed, and a sample was then washed briefly in sterile phosphate-buffered saline (PBS). Samples for RNA or protein extraction were fixed in RNALater for 48 hours and then stored at -80°C.

Mouse model of maternal hypoxia

To assess the effect of maternal hypoxia on SPINT1, placentas were obtained from mothers exposed to hypoxia (10% inspired O_2) or normoxia (21% inspired O_2) from gestational day 14.5-19.5 as previously described[5]. Briefly, virgin C57BL/6 J female mice, aged 6–8 weeks, were housed in groups of two to five per cage under a 12:12 h light:dark cycle at 22°C and were mated overnight with C57BL/6 J males. The presence of a copulatory plug was designated as day (D)1 of pregnancy (term ~D20.5). All animals had *ad libitum* access to water and food [RM3, energy from fat 11%, protein 26%, carbohydrate 62% (simple sugar 7%), 15.3 MJ kg^{-1}, diet code 801066; Special Diet Services, Witham, Essex, UK]. Mated females were weighed daily and the daily consumption of food and water was measured per cage to calculate intake per mouse per day. Pregnant mice were exposed to chronic, normobaric hypoxia for 5 day periods by placing their cages into an isolated PVC chamber (PFI Plastics Ltd, Milton Keynes, UK) in which the oxygen content was reduced to 10% by displacing oxygen with nitrogen using a nitrogen generator (N2MID60; Domnick Hunter Ltd, Warwick, UK). Control mice were maintained at room oxygen (21%). All procedures described were approved by the Ethical Review Committee of the University of Cambridge (Cambridge, UK) and were carried out in accordance with UK Animals (Scientific Procedures) Act 1986 as previously reported [5].

On day 19 of gestation, dams were anesthetised before death (by cervical dislocation) with an intraperitoneal injection of fentanyl-fluanisone and midazolam in sterile water (1:1L2, 10 ug/mL; Janssen animal health). The uterus was removed, and all placentas were dissected free of fetus and membranes, weighed and immediately snap frozen whole in liquid nitrogen and stored at -80C for molecular analyses. For RNA and protein studies, 1 placenta per litter was included.
Proteins were extracted from the second lightest placenta per litter (where possible) in lysis buffer containing 20 mM Tris (pH 7.5), 150 mM NaCl, 1 mM Na_2EDTA, 1 mM EGTA, 1% Triton X-100, 2.5 mM sodium pyrophosphate, 1 mM β-glycerolphosphate, 1 mM Na_3VO_4, and complete mini proteases inhibitor cocktail (Roche Diagnostics, East Sussex, UK). Lysates protein concentration was determined using a Bicinchoninic acid assay (Sigma-Aldrich).
RNA was extracted from the smallest placenta per litter (where possible) using the RNeasy Plus Mini Kit (Qiagen, Machester, UK) and the quantity of RNA determined using a NanoDrop spectrophotometer (NanoDrop Technologies, Inc.). From each sample, 2.5ug of total RNA was reverse transcribed to cDNA using High Capacity cDNA Reverse Transcription Kit with random primers (Applied Biosystems, Paisley,UK).

ELISAs to measure circulating levels of proteins
Commercially available protein-specific ELISAs were obtained and used to measure the concentration of each protein of interest in pg/ml (see table S3). Maternal plasma levels of sFlt-1 and PlGF were each measured with a commercial electrochemiluminescence immunoassay platform (Roche Diagnostics). These assays have received Conformité Européenne marking for use as *in vitro* medical devices.

RT-PCR on human and mouse placenta
To measure *SPINT1* mRNA expression in human and mouse placenta, mRNA was extracted from 20-30 mg of RNA later preserved frozen human placental samples by homogenization or from cytotrophoblast using an RNeasy mini-kit (Qiagen). 1µg of RNA was converted to cDNA using Applied Biosystems high capacity cDNA Reverse Transcriptase Kit (Life Technologies, Carlsbad, CA, USA). Taqman expression assays for human *SPINT1*, Murine *Spint1*, *TOP1, CYC1*, *Ubc*, *Polr2A* were used. For comparisons between human placental samples, data was normalised to the geometric mean of two housekeepers; *TOP1* and *CYC1*. For mouse studies data was normalised to the geometric mean of *Ubc* and *Polr2A*. RT-PCR was performed on the CFX 384 (Biorad, Hercules, CA, USA) using FAM-labeled Taqman universal PCR mastermix (Life Technologies) with the following run conditions: 50 ^{o}C for 2 minutes; 95 ^{o}C for 10 minutes, 95 ^{o}C for 15 seconds, 60 ^{o}C for 1 minute (40 cycles).

Taqman gene expression assays used were as follows:
Taqman Gene Expression Assay, Hs00173678_m1 *SPINT1*
Taqman Gene Expression Assay, Hs00243257_m1 *TOP1*
Taqman Gene Expression Assay, Hs00357717_m1 *CYC1*
Taqman Gene Expression Assay, Mm00444186_m1 *Spint1* (mouse)
Taqman Gene Expression Assay, Mm00839502_m1, *Polr2a*
Taqman Gene Expression Assay, Mm01198158_m1, *Ubc*

Western Blot on human and mouse placenta

To examine SPINT1 protein expression, western blot analysis was undertaken on human and mouse placenta. 20µg of placental lysates (n=23 preterm and n=13 FGR) or 15 µg of trophoblast (n=11 separate isolations) were separated on 10% SDS-polyacrylamide gels with wet transfer to PVDF membranes (Millipore, Billerica, MA). Membranes were blotted overnight with an antibody targeting SPINT1 (Rabbit anti-human SPINT1, Sigma, 1:250), an anti-GAPDH antibody for human placental samples (1:5000, Cell Signaling Technology, Danvers, MA, USA) or an anti-b-actin antibody for trophoblast samples (1:10,000, Sigma) and visualized using the Amersham ECLTM Prime Western blotting detection reagent (VWR International) and ChemiDoc XRS (BioRad, Hercules, CA, USA). Relative densitometry was determined in all samples using Image Lab (BioRad).

For murine placental western blots, 20µg of placental lysates were separated on a 12% SDS-polyacrylamide gel with wet transfer to PVDF membranes (Millipore, Billerica, MA). Membranes were blotted overnight with an antibody targeting murine SPINT1 (R&D systems, 1:2000 dilution) and b-actin followed by an anti-mouse HRP. Protein bands were visulaised using the Amersham ECLTM Prime Western blotting detection reagent (VWR International) and ChemiDoc XRS (BioRad, Hercules, CA, USA). Relative densitometry was determined in all samples using Image Lab (BioRad).

Statistical Analyses

Data summarized as mean (SD), median [25th – 75th percentile], median (minimum, maximum) and number (%) according to distribution. Hypothesis testing between SGA status used Mann-Whitney rank sum test for continuous and Fisher's exact test for categorical data. Predictive performance, presented as point estimate and Wilson based 95% confidence intervals, was assessed using area under receiver operating characteristic curve (AUC area), sensitivity at 90% specificity and positive (PPV) and negative (NPV) at the known prevalence of SGA in this dataset. Multivariate logistic modelling was performed for all combinations of SPINT1, GDF15, DAPK1 and syndecan-1, predictive probability was calculated, and discrimination assessed using area under ROC curves and sensitivity at 90% specificity.

LASSO penalized regression was also performed in analyses adjusting for maternal age, smoking status, parity and BMI. Comparison of area under ROC curves used the DeLong test statistic. For functional data a minimum of technical triplicates were performed for each biological replicate, with a minimum of three biological replicates (each from different patients) performed for each *in-vitro* study. When two groups were analysed a t-test (parametric) or a Mann-Whitney test (non-parametric) was used. For hypoxia data, a Wilcoxon matched pairs-ranks test was used. Significance level was set at 0.05 and no adjustment was made for multiple comparisons. Statistical software used was Stata v15 (StataCorp. 2017. *Stata Statistical Software: Release 15*. College Station, TX: StataCorp LLC) and diagt program (Summary statistics for diagnostic tests. P. T. Seed and A. Tobias. Reprinted in Stata Technical Bulletin Reprints, vol. 10, pp. 90–93., from http://fmwww.bc.edu/RePEc/bocode/d last accessed 1 Nov 2018) or Graphpad Prism 6 (GraphPad Software, LA Jolla, CA).

References for the supplementary material

1 Gardosi, J., Francis A. *Customised Weight Centile Calculator-GROW-Centile v.5.12/6.2*, <www.gestation.net> (2009).

2 Gomez, O. *et al.* Reference ranges for uterine artery mean pulsatility index at 11-41 weeks of gestation. *Ultrasound in obstetrics & gynecology : the official journal of the International Society of Ultrasound in Obstetrics and Gynecology* **32**, 128-132, doi:10.1002/uog.5315 (2008).

3 Dobbins, T. A., Sullivan, E. A., Roberts, C. L. & Simpson, J. M. Australian national birthweight percentiles by sex and gestational age, 1998-2007. *Med J Aust* **197**, 291-294 (2012).

4 Kaitu'u-Lino, T. J. *et al.* Characterization of protocols for primary trophoblast purification, optimized for functional investigation of sFlt-1 and soluble endoglin. *Pregnancy Hypertens* **4**, 287-295, doi:10.1016/j.preghy.2014.09.003 (2014).

5 Higgins, J. S., Vaughan, O. R., Fernandez de Liger, E., Fowden, A. L. & Sferruzzi-Perri, A. N. Placental phenotype and resource allocation to fetal growth are modified by the timing and degree of hypoxia during mouse pregnancy. *The Journal of physiology* **594**, 1341-1356, doi:10.1113/JP271057 (2016).
