# Supplementary material for: Circulating SPINT1 is a biomarker of pregnancies with poor placental function and fetal growth restriction
Source: Nat Commun. 2020 May 15;11:2411. doi: 10.1038/s41467-020-16346-x (PMC7228948; doi:10.1038/s41467-020-16346-x)
Supplement: Supplementary file 3 — Reporting Summary [file 41467_2020_16346_MOESM3_ESM.pdf]

nature research

Corresponding author(s): Tu'uhevaha Kaitu'u-Lino

Last updated by author(s): Mar 5, 2020

Reporting Summary

Nature Research wishes to improve the reproducibility of the work that we publish. This form provides structure for consistency and transparency in reporting. For further information on Nature Research policies, see Authors & Referees and the Editorial Policy Checklist .

Statistics

For all statistical analyses, confirm that the following items are present in the figure legend, table legend, main text, or Methods section.

n/a	Confirmed	
☐	☒	The exact sample size (*n*) for each experimental group/condition, given as a discrete number and unit of measurement
☐	☒	A statement on whether measurements were taken from distinct samples or whether the same sample was measured repeatedly
☐	☒	The statistical test(s) used AND whether they are one- or two-sided<br>*Only common tests should be described solely by name; describe more complex techniques in the Methods section.*
☐	☒	A description of all covariates tested
☐	☒	A description of any assumptions or corrections, such as tests of normality and adjustment for multiple comparisons
☐	☒	A full description of the statistical parameters including central tendency (e.g. means) or other basic estimates (e.g. regression coefficient) AND variation (e.g. standard deviation) or associated estimates of uncertainty (e.g. confidence intervals)
☐	☒	For null hypothesis testing, the test statistic (e.g. *F*, *t*, *r*) with confidence intervals, effect sizes, degrees of freedom and *P* value noted<br>*Give P values as exact values whenever suitable.*
☒	☐	For Bayesian analysis, information on the choice of priors and Markov chain Monte Carlo settings
☒	☐	For hierarchical and complex designs, identification of the appropriate level for tests and full reporting of outcomes
☒	☐	Estimates of effect sizes (e.g. Cohen's *d*, Pearson's *r*), indicating how they were calculated

Our web collection on statistics for biologists contains articles on many of the points above.

Software and code

Policy information about availability of computer code

Data collection	Clinical data collected electronically using Redcap via a license held by University of Melbourne. Image Lab, version 6.0.1 build 34 Standard Edition (c) 2017 Bio-Rad laboratories, Inc. To determine customised birth weight centiles, GROW software (www.gestation.net, v5.12/6.2,) was used.
Data analysis	Statistical software used was Stata v15 (StataCorp. 2017. Stata Statistical Software: Release 15. College Station, TX: StataCorp LLC) and diagt program (Summary statistics for diagnostic tests. P. T. Seed and A. Tobias. Reprinted in Stata Technical Bulletin Reprints, vol. 10, pp. 90–93., from http://fmwww.bc.edu/RePEc/bocode/d last accessed 1 Nov 2018) or Graphpad Prism 6 (GraphPad Software, LA Jolla, CA).

For manuscripts utilizing custom algorithms or software that are central to the research but not yet described in published literature, software must be made available to editors/reviewers. We strongly encourage code deposition in a community repository (e.g. GitHub). See the Nature Research guidelines for submitting code & software for further information.

Data

Policy information about availability of data

All manuscripts must include a data availability statement. This statement should provide the following information, where applicable:

- Accession codes, unique identifiers, or web links for publicly available datasets
- A list of figures that have associated raw data
- A description of any restrictions on data availability

The data that support the findings of this study are available from the corresponding author upon reasonable request.

Field-specific reporting

Please select the one below that is the best fit for your research. If you are not sure, read the appropriate sections before making your selection.

☒ Life sciences ☐ Behavioural & social sciences ☐ Ecological, evolutionary & environmental sciences

For a reference copy of the document with all sections, see nature.com/documents/nr-reporting-summary-flat.pdf

Life sciences study design

All studies must disclose on these points even when the disclosure is negative.

Sample size	As described, this was an exploratory study, not a clinical trial, so a pre-hoc power calculation could not be performed. Our discovery studies were undertaken using a nested case control design, where all cases of SGA were included and matched controls. For validation studies, we measured the expression of analytes in the entire population.
Data exclusions	We did not exclude any data. The entire cohort was samples collected from our prospective cohort was included.
Replication	For the large cohort studies ELISAs were firstly optimised for sample dilution, then used to quantify protein concentrations and did not need to be repeated.<br><br>For the laboratory observation studies on mouse and human placentas we optimised PCR and Western blot techniques and quantified mRNA and protein levels once, with technical replicates included in the RT-PCRs. These studies were not replicated.
Randomization	Not applicable to our study - this was a cohort collection study, and thus there were no treatments or conditions requiring randomization.
Blinding	The technicians performing the ELISAs for the human samples were blinded to the clinical groups for all ELISAs. The technicians were not specifically blinded for the laboratory studies measuring mRNA and proteins in the mouse and human placental samples. However, they did not select the samples, or run the data analyses. Of note, although the technicians were not blinded, samples were run in random order with cases and controls interspersed, reducing any experimental bias.

Reporting for specific materials, systems and methods

We require information from authors about some types of materials, experimental systems and methods used in many studies. Here, indicate whether each material, system or method listed is relevant to your study. If you are not sure if a list item applies to your research, read the appropriate section before selecting a response.

Materials & experimental systems

n/a	Involved in the study	
☐	☒	Antibodies
☒	☐	Eukaryotic cell lines
☒	☐	Palaeontology
☐	☒	Animals and other organisms
☐	☒	Human research participants
☒	☐	Clinical data

Methods

n/a	Involved in the study	
☐	☐	ChIP-seq
☐	☐	Flow cytometry
☐	☐	MRI-based neuroimaging

Antibodies

Antibodies used	Anti-SPINT1 (human), Anti-mouse HAI-1 Antibody
Validation	The human SPINT1 antibody was sourced from sigma aldrich (cat#HPA006903, lot#A40210) - developed and validated by the Human Protein Atlas (HPA) project for Western Blot<br>The mouse HAI-1 antibody (cat#AF1141, clone RDSAF1141SP) was sourced from R&D Systems. Referenced by Hoskiko et al, Cancer Res, 2013; 73 (8): 2658-70. It has been validated for immunohistochemistry, immunoprecipitation and Western blot by RnD systems.<br>The anti-GAPDH antibody (cat#3683, clone 14C10) is a Rabbit mAb from Cell signaling Tech. It has been validated for Western Blot by Cell Signaling Tech.<br>The anti-actin antibody (cat#sc-8432, clone C-2) is a mouse monoclonal IgG obtained from Santa Cruz. It has been validated by Santa Cruz for Western Blot

Animals and other organisms

Policy information about studies involving animals; ARRIVE guidelines recommended for reporting animal research

Laboratory animals	C57BL/6J mice were used. Female mice were mated with C57BL/6J male studs at 6-8wks of age and placentas collected.
Wild animals	No wild animals were used in this study
Field-collected samples	No field collected samples were used in this study
Ethics oversight	All procedures described were approved by the Ethical Review Committee of the University of Cambridge (Cambridge, UK) and were carried out in accordance with UK Animals (Scientific Procedures) Act 1986

Note that full information on the approval of the study protocol must also be provided in the manuscript.

Human research participants

Policy information about studies involving human research participants

Population characteristics

There were three cohorts:

A) FLAG (Fetal Longitudinal Assessment of Growth) cohort - large prospective cohort (1996) participants.

This was a large prospective collection of unselected women presenting for pregnancy care at Mercy Hospital for Women, Victoria, Australia. Hence population characteristics:

- All were pregnant women
- Unselected population - we approached women presenting around 28 weeks gestation attending their oral glucose tolerance test for gestational diabetes
- We kept all participants in the study, irrespective of the pregnancy complications they developed

The detailed clinical information of the cohort are precisely described in supplementary tables S1 and S2.

B) MAVIS (Manchester Antenatal Vascular Service) cohort - Case control study of 291 women in Manchester UK presenting to a high risk clinic

Women cared for in the MAVIS clinic are those with hypertension during the current pregnancy, or had a prior pregnancy with hypertension. They are at higher risk of developing small for gestational age pregnancies arising from placental insufficiency. Hence, the population characteristics:

- All were pregnant women
- A selected, defined population - hypertension in the current pregnancy, or in a prior pregnancy
- We kept all participants irrespective of the pregnancy complications they developed

The detailed clinical information of both the case/control cohort of 291 participants, and the entire MAVIS cohort from which these samples were collected from are shown in supplementary table S5.

C) Case control cohort of women with preterm fetal growth restriction, or controls

These women were recruited at Mercy Hospital for women. Hence population characteristics:

- All were pregnant women
- They were either diagnosed with preterm fetal growth restriction (delivered <34 weeks gestation), or not (controls)

Recruitment

1) FLAG Cohort:

Unselected pregnant women presenting for their oral glucose tolerance test around 28 weeks' gestation (to test for gestational diabetes mellitus) were invited to participate. We did restrict the cohort to english-speaking women (so we can be sure they understood the informed written consent) and aged over 18 years. But there were no other restrictions and we believe the risk of bias is minimal.

2) MAVIS Cohort

All pregnant women presenting the MAVIS clinic for care were invited to participate in this prospective observational research study (as long as they are age 18+, and able to understand English). Other than representing a high risk cohort (pregnant women with vascular disease at at higher risk of developing placental insufficiency), we believe there is also minimal risk of bias.

3) Case control cohort of women with preterm fetal growth restriction, or controls.

These were samples collected as part of an ongoing biobank collection at Mercy Hospital for Women. Women with preterm fetal growth restriction were invited to participate were bloods were collected and, where possible, the placenta at the time of birth. Collections were also done for control, healthy pregnant women who were willing to donate their samples for research. Again, women had to be 18+ and able to understand english.

The samples were collected in a uniform manner, and contemporaneously at the same hospital. The samples were chosen, then SPINT1 assays run in batch. There is no obvious self-selection bias, as women were consented and enrolled, before their clinical phenotype (control, FGR or SGA) determined at a later timepoint after clinical characterisation.

Ethics oversight: Human Ethics approval was obtained for this study from the Mercy Health Human Research Ethics Committee (R14/12, R11/34)

Note that full information on the approval of the study protocol must also be provided in the manuscript.

ChIP-seq

Data deposition

- [ ] Confirm that both raw and final processed data have been deposited in a public database such as GEO.
- [ ] Confirm that you have deposited or provided access to graph files (e.g. BED files) for the called peaks.

Data access links
May remain private before publication.: *For "Initial submission" or "Revised version" documents, provide reviewer access links. For your "Final submission" document, provide a link to the deposited data.*

Files in database submission: *Provide a list of all files available in the database submission.*

Genome browser session
(e.g. UCSC): *Provide a link to an anonymized genome browser session for "Initial submission" and "Revised version" documents only, to enable peer review. Write "no longer applicable" for "Final submission" documents.*

Methodology

Replicates: *Describe the experimental replicates, specifying number, type and replicate agreement.*

Sequencing depth: *Describe the sequencing depth for each experiment, providing the total number of reads, uniquely mapped reads, length of reads and whether they were paired- or single-end.*

Antibodies: *Describe the antibodies used for the ChIP-seq experiments; as applicable, provide supplier name, catalog number, clone name, and lot number.*

Peak calling parameters: *Specify the command line program and parameters used for read mapping and peak calling, including the ChIP, control and index files used.*

Data quality: *Describe the methods used to ensure data quality in full detail, including how many peaks are at FDR 5% and above 5-fold enrichment.*

Software: *Describe the software used to collect and analyze the ChIP-seq data. For custom code that has been deposited into a community repository, provide accession details.*

Flow Cytometry

Plots

Confirm that:

- [ ] The axis labels state the marker and fluorochrome used (e.g. CD4-FITC).
- [ ] The axis scales are clearly visible. Include numbers along axes only for bottom left plot of group (a 'group' is an analysis of identical markers).
- [ ] All plots are contour plots with outliers or pseudocolor plots.
- [ ] A numerical value for number of cells or percentage (with statistics) is provided.

Methodology

Sample preparation: *Describe the sample preparation, detailing the biological source of the cells and any tissue processing steps used.*

Instrument: *Identify the instrument used for data collection, specifying make and model number.*

Software: *Describe the software used to collect and analyze the flow cytometry data. For custom code that has been deposited into a community repository, provide accession details.*

Cell population abundance: *Describe the abundance of the relevant cell populations within post-sort fractions, providing details on the purity of the samples and how it was determined.*

Gating strategy

Describe the gating strategy used for all relevant experiments, specifying the preliminary FSC/SSC gates of the starting cell population, indicating where boundaries between "positive" and "negative" staining cell populations are defined.

☐ Tick this box to confirm that a figure exemplifying the gating strategy is provided in the Supplementary Information.

Magnetic resonance imaging

Experimental design

Design type	*Indicate task or resting state; event-related or block design.*
Design specifications	*Specify the number of blocks, trials or experimental units per session and/or subject, and specify the length of each trial or block (if trials are blocked) and interval between trials.*
Behavioral performance measures	*State number and/or type of variables recorded (e.g. correct button press, response time) and what statistics were used to establish that the subjects were performing the task as expected (e.g. mean, range, and/or standard deviation across subjects).*

Acquisition

Imaging type(s)	*Specify: functional, structural, diffusion, perfusion.*
Field strength	*Specify in Tesla*
Sequence & imaging parameters	*Specify the pulse sequence type (gradient echo, spin echo, etc.), imaging type (EPI, spiral, etc.), field of view, matrix size, slice thickness, orientation and TE/TR/flip angle.*
Area of acquisition	*State whether a whole brain scan was used OR define the area of acquisition, describing how the region was determined.*

Diffusion MRI ☐ Used ☐ Not used

Preprocessing

Preprocessing software	*Provide detail on software version and revision number and on specific parameters (model/functions, brain extraction, segmentation, smoothing kernel size, etc.).*
Normalization	*If data were normalized/standardized, describe the approach(es): specify linear or non-linear and define image types used for transformation OR indicate that data were not normalized and explain rationale for lack of normalization.*
Normalization template	*Describe the template used for normalization/transformation, specifying subject space or group standardized space (e.g. original Talairach, MNI305, ICBM152) OR indicate that the data were not normalized.*
Noise and artifact removal	*Describe your procedure(s) for artifact and structured noise removal, specifying motion parameters, tissue signals and physiological signals (heart rate, respiration).*
Volume censoring	*Define your software and/or method and criteria for volume censoring, and state the extent of such censoring.*

Statistical modeling & inference

Model type and settings	*Specify type (mass univariate, multivariate, RSA, predictive, etc.) and describe essential details of the model at the first and second levels (e.g. fixed, random or mixed effects; drift or auto-correlation).*
Effect(s) tested	*Define precise effect in terms of the task or stimulus conditions instead of psychological concepts and indicate whether ANOVA or factorial designs were used.*

Specify type of analysis: ☐ Whole brain ☐ ROI-based ☐ Both

Statistic type for inference (See Eklund et al. 2016)	*Specify voxel-wise or cluster-wise and report all relevant parameters for cluster-wise methods.*
Correction	*Describe the type of correction and how it is obtained for multiple comparisons (e.g. FWE, FDR, permutation or Monte Carlo).*

Models & analysis

n/a	Involved in the study
☐	☐ Functional and/or effective connectivity
☐	☐ Graph analysis
☐	☐ Multivariate modeling or predictive analysis

Functional and/or effective connectivity	*Report the measures of dependence used and the model details (e.g. Pearson correlation, partial correlation, mutual information).*
Graph analysis	*Report the dependent variable and connectivity measure, specifying weighted graph or binarized graph, subject- or group-level, and the global and/or node summaries used (e.g. clustering coefficient, efficiency, etc.).*
Multivariate modeling and predictive analysis	*Specify independent variables, features extraction and dimension reduction, model, training and evaluation metrics.*
